# Calcium phosphate-based nanomedicine mediated CRISPR/Cas9 delivery for prostate cancer therapy

**DOI:** 10.3389/fbioe.2022.1078342

**Published:** 2022-12-14

**Authors:** Chao-Gang Wei, Rui Zhang, Lan-Yi Wei, Peng Pan, He Zu, Ya-Zhen Liu, Yong Wang, Jun-Kang Shen

**Affiliations:** ^1^ Department of Radiology, The Second Affiliated Hospital of Soochow University, Suzhou, China; ^2^ State Key Laboratory of Radiation Medicine and Protection, School of Radiation Medicine and Protection, School for Radiological and Interdisciplinary Sciences (RAD-X), Collaborative Innovation Center of Radiation Medicine of Jiangsu Higher Education Institutions, Soochow University, Suzhou, China; ^3^ Department of Emergency, The Second Affiliated Hospital of Soochow University, Suzhou, China

**Keywords:** CRISPR/Cas9, *EphA2*, nanomedicine, non-viral carrier, target delivery, castration-resistance prostate cancer

## Abstract

**Introduction:** Erythropoietin producing hepatocyte receptor A2 (*EphA2*) is widely presented in the tumor cells, closely related to tumor cell migration, not cell apoptosis and proliferation. Based on its high expression in castration-resistant prostate cancer (CRPC), we herein develop a CRISPR-Cas9-based genome-editing nanomedicine to target erythropoietin producing hepatocyte receptor A2 for the treatment of castration-resistant prostate cancer.

**Methods:** To this end, TAT was designed to stabilize the distribution of calcium, and then bound to ribonucleoprotein (RNP) to form nanoparticles RNP@CaP-TAT.

**Results:** This nanoparticle has a simple synthesis process with good biocompatible, to achieve the knockout of tumor cells (PC-3) targeting erythropoietin producing hepatocyte receptor A2 gene and to effectively suppress the migration of tumor cells.

**Discussion:** This delivery genome editing system provides a promising gene therapy strategy for the treatment of castration-resistant prostate cancer, showing good potential against castration-resistant prostate cancer tumor metastasis. In addition, it can be extended to other types of cancer with highly heterogeneous gene expression.

## 1 Introduction

CRISPR-Cas9 system have been developed and validated as powerful and precise tools for gene editing, broadly used in a variety of cell line- and embryo-based experiments (Platt et al., 2014; Wang et al., 2017). CRISPR-Cas9-mediated somatic genome editing has shown great potential in treating various malignant tumors in animal models, such as Colorectal Cancer (Wan et al., 2021). Castration-resistant prostate cancer (CRPC) is a progressive stage of most patients despite accepted medical or surgical castration therapy (Chandrasekar et al., 2015). Currently, several novel treatments of CRPC, including new cytostatic agents, second-generation antiandrogens, immunotherapy, and radioisotopes, have been investigated (Henriquez et al., 2021). However, few studies reported the gene therapeutic strategy about CRPC. Erythropoietin-producing hepatocellular receptor A2 (*EphA2*), as a key member of the receptor tyrosine kinase (*RTK*) family, can regulate oncological behaviors of cancer cells through different signaling pathways (Markosyan et al., 2019; Mao et al., 2021), including CRPC. Based on its high expression in human CRPC, we herein intend using CRISPR platform to achieve knockout of *EphA2* to slow tumor progression.

To be therapeutically effective, the efficient delivery of CRISPR-Cas9 cargos into cell nucleus with the desired concentration is critical. At present, the commonly delivery method is RNP delivery, which represents as the protein form of CRISPR-Cas9 system. Non-viral delivery modes have attracted numerous attentions, largely owing to their favorable biocompatibility, tailored biophysical properties and flexible packing capabilities, such as liposomes (Chang et al., 2019; Liu et al., 2019), gold nanoparticles (Lee et al., 2018), polymers (Anderson et al., 2003; Wan et al., 2020; O'Keeffe Ahern et al., 2022) and metal-organic frameworks (MOFs) (Lei et al., 2022). Over the past few decades, nanomedicine has witnessed the large evolution from biologically inert entities to more intelligent fields, which aimed to improve *in vivo* functionality (Li and Kataoka, 2021). However, it is still controversial that about these carriers’ toxicity, complex synthesis process and metal residue, thus a RNP coated platform with simple synthesis process, better biocompatibility and high delivery efficiency needs to be developed.

Calcium phosphate-based biomaterials have been well studied in biomedical fields due to their outstanding chemical and biological properties (Qi et al., 2019; Zhou et al., 2020), but its poor dispersibility and broad particle size distribution seriously affect its endocytosis efficiency. Cell-penetrating peptides (CPPs), promising component of non-viral gene therapy, can mediate the transmembrane transport (Ishiguro et al., 2017). TAT, a particular type of CPP, involved in the replication of human immunodeficiency virus type 1 (HIV-1), has been proven to possess unusual translocation capabilities to cross biological membrane directly, independent of the intracellular environment and physical factors (Vives et al., 1997). Previous studies have been reported that its particle size and transfection efficiency are affected by mixing CaCl_2_ and TAT/pDNA complex solution directly. In view of these merits, we hypotheses that modification of cationic calcium with TAT to stabilize cationic calcium, avoid aggregation, and improve transfection efficiency.

Herein, we report a Calcium phosphate-based nanomedicine delivery strategy for RNP that can efficiently target *EphA2* mutation for CRPC gene therapy. We first obtain RNP, by combining Cas9 endonuclease with one stand of single guide RNA (sgRNA), which is designed to target *EphA2* genomic locus. After mixing CaCl_2_ and TAT, Cas9 RNP is further complexed with compound (termed as Ca-TAT) to form delivery nanoparticles, abbreviated as “RNP@CaP-TAT”. This nanoparticle is formed with calcium phosphate as the core, with good function of delivery and biosafety, and has been shown to effectively inhibit cell migration *in vitro*, thereby paving a safe way for the next *in vivo* therapeutic genome editing towards CRPC gene therapy, especially in the inhibition of CRPC tumor metastasis.

## 2 Materials and methods

### 2.1 Materials

Cas9 protein was from Sino Biological (Beijing, China), and sgRNA was synthesized in House. TAT peptide (GRKKRRQRRRAHQN) was from Sangon Biothch (Shanghai, China). Calcium chloride (CaCl_2_) was supplied by Aladdin Industrial Corporation. LysoTracker Red (DND-99) and Wheat Germ Agglutinin (WGA) were bought from Invitrogen (CA, United States), and Hochest 33342 was from Beyotim (Shanghai, China), Cas9 labeled EGFP was obtained from Novoprotein (Suzhou, China). PC-3 cells were obtained from the Type Culture Collection of the Chinese Academy of Sciences (Shanghai, China). The cell culture medium (Ham’s F-12k Nutrient Mixture, Kaighn’s modified with L-glutamine) was purchased through Procell (Wuhan, China). Fetal bovine serum (FBS), Penicillin-streptomycin and Trypsin-EDTA was purchased through Gibco. EphA2 (D4A2) Rabbit mAb was from Cell Signaling Technology (Massachusetts, United States). Cell Counting kit (CCK)-8 assay were purchased from Dojindo Molecular Technologies, Inc. (Kumamoto, Japan). Other reagents were purchased from Beyotime (Shanghai, China) (Van Asbeck et al., 2013), and Deionized water was purified in-house using an Evoqua Ultra Clear Glass Panel Systems (Germany) and was used for all experiments.

### 2.2 Plasmid construction

The lentiCRISPR v2 was a gift from Feng Zhang (Addgene plasmid # 52961; http://n2t.net/addgene:52961; RRID:Addgene_52961) (Sanjana et al., 2014). The plasmid was enzymatically digested by BsmBI to form a sticky end notch in gRNA scaffold. Followed, a pair of compatible and annealed oligos (Supplementary Figure S1A) were ligated with the backbone of the vector by T4 ligase. The ligation product was added to the competent cell suspension and transferred onto LB medium containing ampicillin. A clonal colony was selected for DNA sequencing to ensure the correct sequence of the ligation fragment. Then the LentiCRISPRv2-EphA2 plasmid was obtained through plasmid extraction kit after amplifying by shaking bacteria.

### 2.3 Transcription of sgRNA *in vitro*

LentiCRISPRv2-EphA2 plasmid was linearized through PCR to obtain sgRNA transcription template. The PCR primers contained T7 transcriptase recognition site, and the TranscriptAid T7 High Yield Transcription Kit (thermo scientific) was used for *in vitro* transcription with the sgRNA transcription template as the substrate. The transcription product was verified by Gel electrophoresis. Supplementary Figure S1B is the sequence of the PCR primers to linearize the sgRNA transcription template.

### 2.4 Preparation and characterization of RNP@CaP-TAT

Ca-TAT complex was synthesized by adding 20 μl of Cacl_2_ solution (0.6 M) to 21.2 μl of TAT solution (0.7 mM)and incubated at room temperature for 1 h after fast pipetting for 20 s; At that point, Cas 9 and sgRNA were mixed at a molar ratio of 1:1 in the DI water by fast pipetting and incubated at room temperature for 30 min. Finally, the RNP@CaP-TAT nanoparticles was obtained, and the total volume was 78.28 µl. After preparing the nanoparticles, they were stored at 4°C for 1–2 h. The resulting product was collected by centrifugation and washed with DI water to remove any residues. The supernatant was collected to calculate the loading capacity and encapsulation efficiency. The nanoparticles were prepared immediately before each experiment.

### 2.5 Assembly of RNP@CaP-TAT

The size and Zeta potential of RNP@CaP-TAT were performed using a dynamic light scattering (Malvern Zetasizer nano-ZS90, Malvern instruments, United Kingdom) at 25°C at pH 7 in aqueous solutions. Transmission electron microscopic (TEM) images were obtained using a Tecnai G2 spirit BioTwin transmission electron microscope (FEI, America) with a voltage of 120 kV. For visualization by TEM, samples were prepared by dropping a solution of production on a copper grid 300 mesh (Electron Microscopy Sciences, LC 300-Cu) and dried in air at ambient condition.

### 2.6 Circular dichroism (CD) analysis of nanoparticles

The nanocomplexes were analyzed by circular dichroism spectrometer (CD/J-815, Japan) with the final protein concentration is 100 μg/ml. Free Cas9 protein, RNP, RNP@CaP (Cas9 protein was 37.2 μg) was tested as a control.

### 2.7 Loading capability characterization

To evaluate the encapsulation efficiency of the RNP, RNP@CaP-TAT was isolated by centrifugation at 4°C, 10,000 rpm for 25 min. The concentrations of RNP complex in the supernatant were measured by a UV–Vis–NIR spectrophotometer (UV-3600, Shimadzu, Japan) at a given wavelength (RNP: 260 nm). The encapsulation efficiency could be calculated according to the equation

$$EE\;\left(\%\right)=\left(C_{1}-C_{2}\right)/C_{1}\times 100\%,$$ (1)

where C_1_ represents the original concentration of RNP in the solution and C_2_ represents the unbound RNP in the supernatant.

### 2.8 Nanoparticles pH sensitivity determination

The pH response of nanoparticles was characterized by DLS which were placed in acetate buffer solutions and cell culture medium with pH 5.0, pH 6.5, and pH 7.4, respectively. After 5 min, 1, 2 and 6 h, DLS was used to measure the particle size distribution.

### 2.9 Agarose gel electrophoresis

The nanoparticles were prepared as defined previously and subsequently, 4 µl of dsDNA containing sgRNA target was added to the nanoparticles or control samples. Then, the DNA Loading Dye was added to the mixture. The mixture of the solutions was loaded onto a 1% agarose gel, and electrophoresed for 30 min at 110 V. An RNA marker was used as a reference marker. The electrophoresis was performed at 110 V and visualized under UV light.

### 2.10 Cell Culture

PC-3 cell lines were grown in F-12K Nutrient Mixture media (Kaighn’s modified with L-glutamine; Mediatech, Inc., Manassas, VA, United States), Dulbecco’s Modified Eagle’s Medium (DMEM; Invitrogen, Grand Island, NY, United States) (for 3T3 cell lines) with 1% (v/v) Penicillin/Streptomycin and 10% (v/v) fetal bovine serum (FBS) at 37°C in 5% CO_2_ humidified air.

### 2.11 Cytosolic Cas9 RNP delivery

PC-3 cells were cultured in glass bottom cell culture dishes at a density of 1 × 10^5^ cells before cytosolic protein delivery. EGFP labeled Cas 9 (EGFP-Cas9) was used to track the uptake of the RNP. Transfected cells incubated for 1, 3, 6 and 12 h. After the specified treatments, the cells were further incubated with fresh L-15 medium containing 10% FBS. The cell nuclei were further stained with Hochest 33,342 following the manufacturer’s instructions. The lysosome was stained by Lysotracker Red according to the manufacturer’s instructions. The cytomembrane was stained by WGA following the manufacturer’s instructions before confocal imaging. Samples at 6 h were also used for western blot detection.

### 2.12 CCK-8 assay

PC-3 and 3T3 cells were inoculated in 96-well plates with 3 × 10^3^ cells per well in 100 μl complete medium. The cells were incubated with different concentrations (250, 200, 150, 100 and 50 μg/ml) of RNP@CaP-TAT nanoparticles for 4 h. Then a standard CCK-8 assay was used to determine the cell proliferation rate. The experiment was performed independently in triplicate.

### 2.13 Western blot

The treated PC-3 cells were lysed in RIPA buffer (Beyotime, China). The lysates were separated by 10% SDS-PAGE gel (Epizyme, China) and transferred onto polyvinylidene fluoride (PVDF) transfer membrane (Millipore, United States). The PVDF membrane was incubated with primary antibody against EphA2 (Rabbit mAb #4513, Cell Signaling Technology) at dilution of 1:1000, and then incubated in the secondary antibody for 1 h at room temperature. The protein bands were detected using an Enhanced Chemiluminescence (ECL) detection kit (Epizyme, China) and quantified using an Image J software (National Institutes of Health, Bethesda, MD, United States). β-actin was usually used as a loading control.

### 2.14 Sanger sequencing

After treating with nanoparticles, cells were collected to extract the DNA using a TIANamp Blood DNA Kit (TIANGEN, Bejing). Then genomic DNA was transcribed with two pairs of specific PCR primers near the cleavage sites to obtain substrates. PCR samples were used to perform Sanger sequencing.

### 2.15 Cell proliferation assay

A Cell Counting kit (CCK)-8 assay (Dojindo Molecular Technologies, Inc., Kumamoto, Japan) was used to assess the proliferation of the transfected cells. The cells were seeded at an initial density of 5 × 10^4^ cells/mL in 96-well plates (6 wells/group) and incubated for 24, 48, 72 and 96 h treated with RNP@CaP-TAT. For the analysis, 10 µl CCK-8 reagent was added to each well, and the plates were incubated for 2 h at 37°C in a 5% CO_2_ incubator. The optical density at 450 nm was then measured. All experiments were performed in triplicate, and the mean results were calculated.

### 2.16 Measurement of cellular apoptosis

Cellular apoptosis was detected using an Annexin V-allophycocyanin (APC)/7-amino-actinomycin D (7-ADD) kit (KeyGENE, China). In this study, flow cytometry was used to analyze cellular apoptosis. The RNP@CaP-TAT and RNP@CaP-TAT-control cell samples were re-suspended with 500 µl 1 × binding buffer at a concentration of (1–3) × 10^6^ cells/ml. Subsequently, Flow cytometry was routinely performed according to the manufacture’s protocol. After incubation with reagents from the indicated assay kit, cells were analyzed using a Beckman cytometer (Beckman Coulter, United States) and CytExpert software (Beckman Coulter, United States). The cells were divided into four regions (Q1–Q4). Region Q1 was representative of mechanical error; region Q2 was representative of late apoptotic or necrotic cells; region Q3 was representative of early apoptotic cells; and region Q4 was representative of living cells.

### 2.17 Wound-healing assay

The cells were seeded in six-well plates. When the cell confluence reached 100%, scratches were made with a 200 μl pipette tip, and cells were washed three times with PBS to remove the delineated cells. The treatments were added to each group. Various therapeutic agents were added to the treated six-well plates and cultured for 48 h in serum-free medium and the images were captured using an inverted microscope (AMG, United States). Plates were imaged (×10 magnification) every 12 h and quantified using an ImageJ software (National Institutes of Health, Bethesda, MD, United States). The cell migration rate (%) = (wound areas at 0 h − wound areas at n h)/wound areas at 0 h.

### 2.18 Transwell migration assay

After the cells were treated with various therapeutic agents for 48 h, the cell migration was studied by the transwell assay as detailed in the Supporting Information. 600 μl 10% serum medium were added to the lower chamber, and 100 μl cell suspension was added to the upper chamber. After 24 h incubation at 37°C, cells were fixed with 4% paraformaldehyde for 30 min and stained with crystal violet for 20 min. The cells on the inserts were wiped off with a cotton swab. Then, the invaded cells were photographed under an inverted light microscope (ZEISS, Germany) at ×200 magnification and counted.

### 2.19 Statistical analysis

Comparisons between different treatments were made using one-way ANOVA. *p < 0.05, **p < 0.01, and ***p < 0.001.

## 3 Results

### 3.1 Acquisition of sgRNA *in vitro*

According to the manufacturer’s protocol, LentiCRISPRv2 was used as the framework to insert the gRNA sequence targeting *EphA2*, and the anglicized sgRNA was obtained by *in vitro* transcription. PCR results showed that the size of the transcription product was about 100 bp (Supplementary Figure S1). To verify the availability of sgRNA transcription *in vitro*, we evaluated the ability of sgRNA cleavage, showing that the sgRNA could effectively and stably knockout the target DNA fragment (Supplementary Figure S2).

### 3.2 Preparation and characterization of RNP@CaP-TAT

**SCHEME 1:**
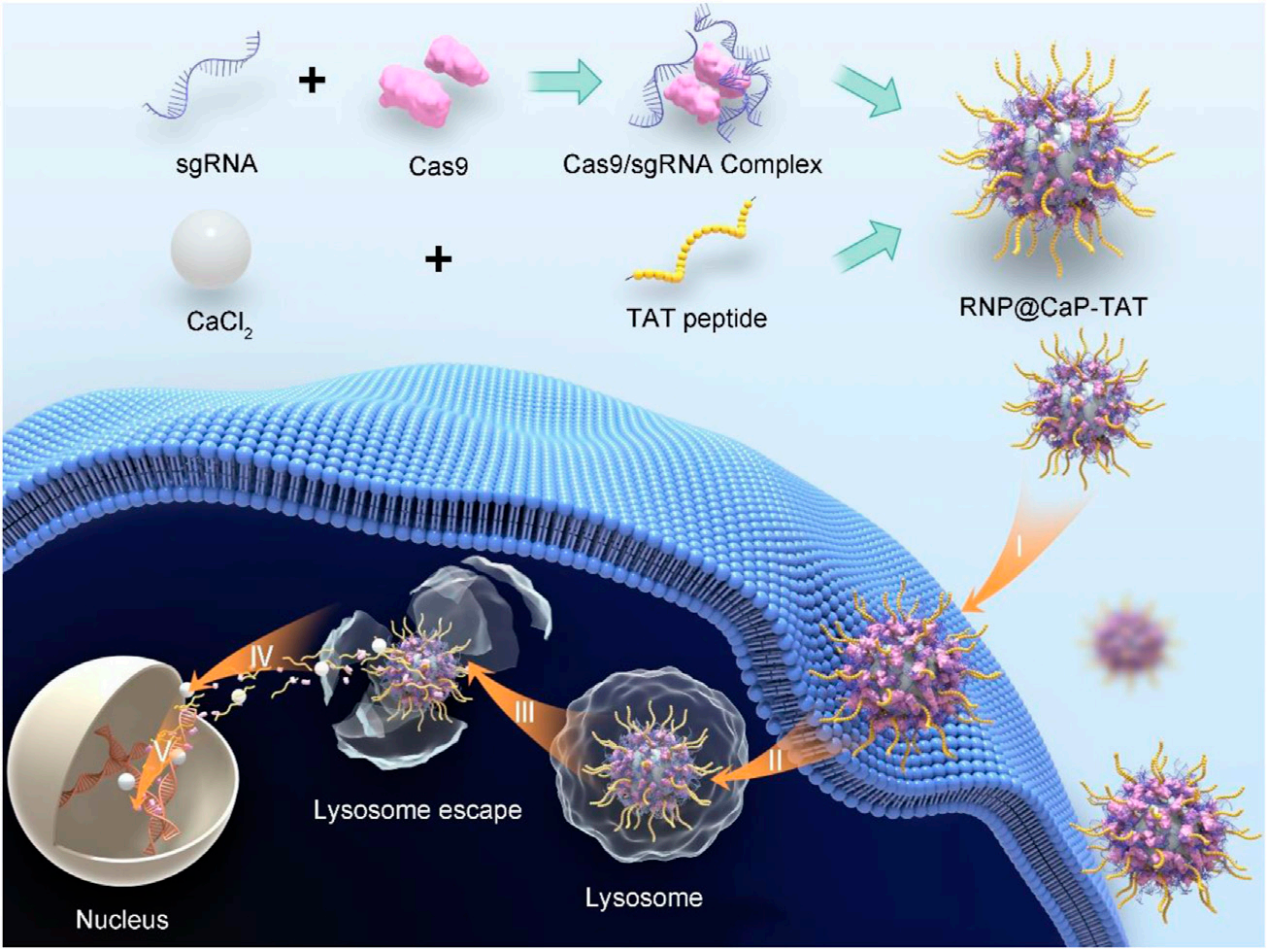
Schematic Illustration of RNP@CaP-TAT Nanopatricles. Preparation of RNP@CaP-TAT and Delivery of Cas9/sgRNA by the non-viral delivery carrier to the nucleus of tumor cells for genome editing. I) Binding to the cell membrane; II) endocytosis; III) Lysosome escape; IV) transport into the nucleus; V) search for the target DNA locus in the chromosome and introduction of double strand breaks for genome editing.

To develop the genome-editing system for the disruption of *EphA2*, firstly we mix the calcium chloride solution and TAT peptides for about 1 h to get the Ca-TAT complex. The RNP complex was formed by mixing the sgRNA targeting *EphA2* and Cas9 protein in water for 30 min. And then, these two complex solutions were put together to form the final nanoparticles, RNP@CaP-TAT (Scheme 1).

**FIGURE 1:**
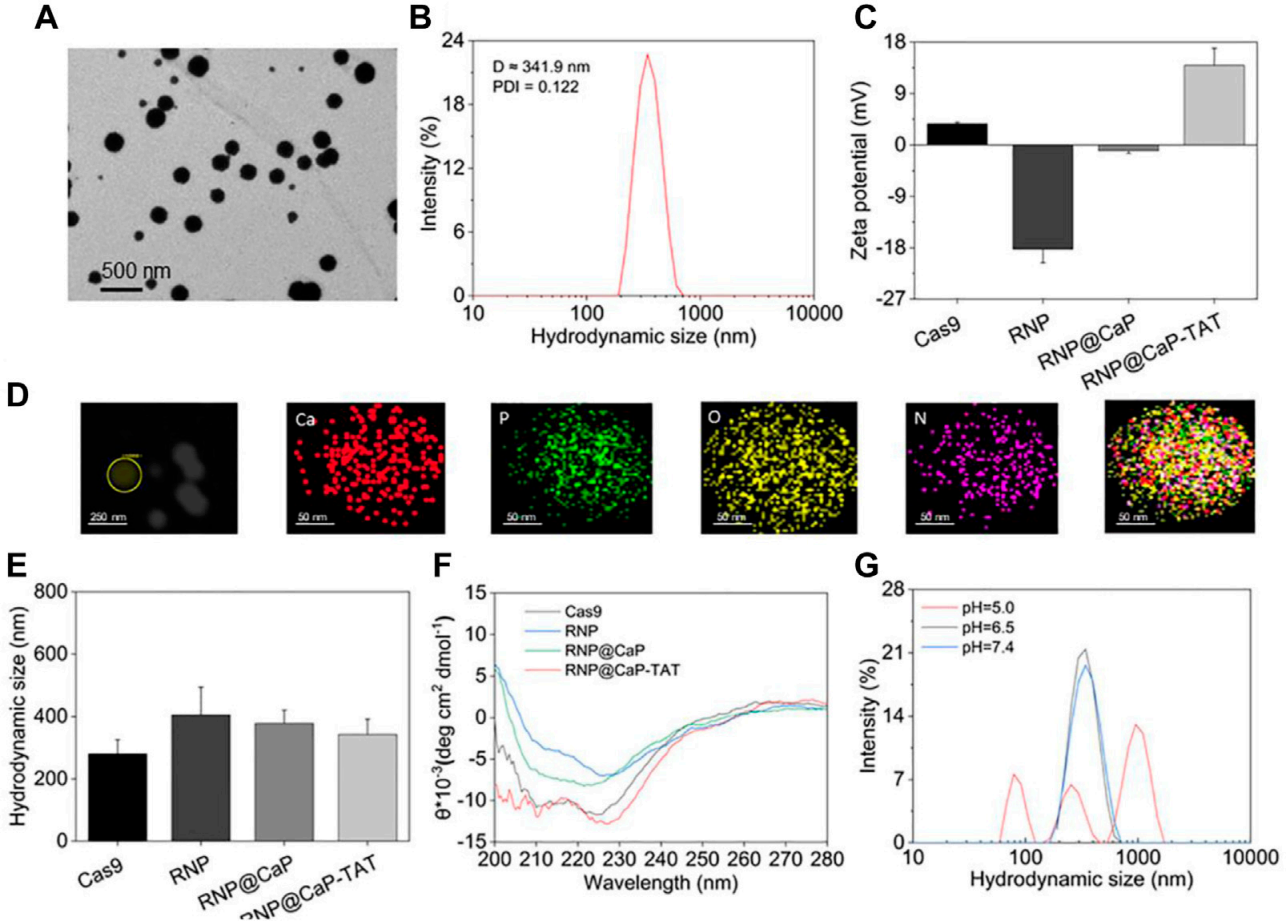
Characterization of RNP@CaP-TAT NPs. **(A)**TEM images of RNP@CaP-TAT NPs. **(B)** Hydrodynamic particle size distribution of RNP@CaP-TAT. **(C)** Zeta Potential of free Cas9, RNP, Ca-TAT complex, RNP@CaP and RNP@CaP-TAT. Bars represent mean ± SD (*n* = 3). **(D)** TEM mapping images of RNP@CaP-TAT. **(E)** DLS analysis of free Cas9, RNP, Ca-TAT complex, RNP@CaP and RNP@CaP-TAT. Bars represent mean ± SD (*n* = 3). **(F)** CD spectra of free Cas9, RNP, Ca-TAT complex, RNP@CaP and RNP@CaP-TAT. **(G)** Hydrodynamic particle size distribution of the RNP@CaP-TAT NPs at pH 7.4, pH 6.5, and pH 5.0.

Due to the large size of calcium core and RNP complex, the mass ratio between them is a key parameter in the synthesis process of this nanoparticle. Proper hydrated particle size was constructed with a 40:1 calcium/RNP mass ratio (Supplementary Figure S3A), and strong reproducibility (Supplementary Figures S3C–F) and satisfactory Zeta potential (Supplementary Figure S3B) can be observed at the same ratio. Transmission electron microscope (TEM) results showed that there is a strong binding affinity with the RNP and Ca-TAT solution to form spherically shaped, stable, and well-dispersible nanoparticles with the diameter of about 190–230 nm (Figure 1A). In addition, the dynamic light scattering (DLS) revealed that the average particle size of RNP@CaP-TAT was around 341.9 nm (Figure 1B) with the zeta potential of approximately +13.97 mV, conforming to the basic requirements for nanoparticles to enter the bilayer of cellular phospholipids. The minor size difference between TEM and DLS may be due to the extension of silk fibroin on the surface of Cas9 protein. And the dynamic change of zeta potential with different compositions fully illustrated the effective synthesis of each component (Figure 1C). During the synthesis process, RNP complex (−18.26 mV) was formed with Cas9 (+3.7 mV) and sgRNA, then combined with positively charged calcium ions to form RNP@CaP (−1.03 mV), and finally constructed positive potential RNP@CaP-TAT nanoparticles with TAT peptide. Elemental mapping indicated that RNP@CaP-TAT consists of calcium, phosphorus, oxygen, and nitrogen (Figure 1D), representing core calcium, Cas9 protein, and phospholipids (phosphate backbone in sgRNA) respectively. The increase in hydrated particle size due to the combination of different components was illustrated by DLS, which is also confirmed the successful assembly of each component (Figure 1E). These results confirmed the formation of RNP@CaP-TAT nanoparticles.

At the wavelength of 200–280 nm and the temperature of 25°C, circular dichroism (CD) results in Figure 1F confirmed that no significant changes in protein secondary structures after RNP were mixed with each component complex, as compared with free Cas9. This result indicated that the synthesis of nanoparticles would not affect the nuclease activity of Cas9, which was crucial for CRISPR/Cas9-mediated knockout effect following the nucleus entry.

DLS was further used to detect the pH response of RNP@CaP-TAT. As shown in Figure 1G, after RNP@CaP-TAT were stored under the three acidic conditions of pH 7.4, pH 6.5, and pH 5.0, the hydration particle size distribution demonstrated that the nanoparticle at both pH 7.4 and pH 6.5 conditions (tumor microenvironment (TEM) acidity) were stable, whereas at pH 5.0, the structure was destroyed. More details can be seen in Supplementary Figure S4. When the incubation time prolonged to 6 h, the hydrated particle size still had no significant change at pH 7.4 and pH 6.5, which is consistent with the TEM results. These results indicated that, RNP@CaP-TAT have the characteristics of functional pH-responsiveness in the lysosome environment of pH 5.0, which promises to break through the capture of lysosomes. While the stable hydrated particle size at pH 6.5 and pH 7.4 indicates RNP@CaP-TAT was stable and would not release RNP early, which enhanced the probability of effective incorporation of RNP into the nucleus.

In addition, after purified by centrifugation, the concentration of Cas9 RNP in the supernatant and RNP in the original solution was detected and calculated by an ultraviolet spectrophotometer (Supplementary Figure S5), the Cas9 RNP loading rate of RNP@CaP-TAT reached 74%. In conclusion, we have synthesized homogeneous and stable RNP nanoparticles with good biological characteristics and high loading efficiency, which provides a reliable basis for subsequent gene editing experiments of CRISPR-Cas9.

### 3.3 Endocytosis and delivery of RNP@CaP-TAT

The cytocompatibility of RNP@CaP-TAT delivery system has been evaluated. Firstly, we assessed its effect on cell viability, showing that the nanoparticles are almost non-toxic at high concentrations (Supplementary Figure S6).

**FIGURE 2:**
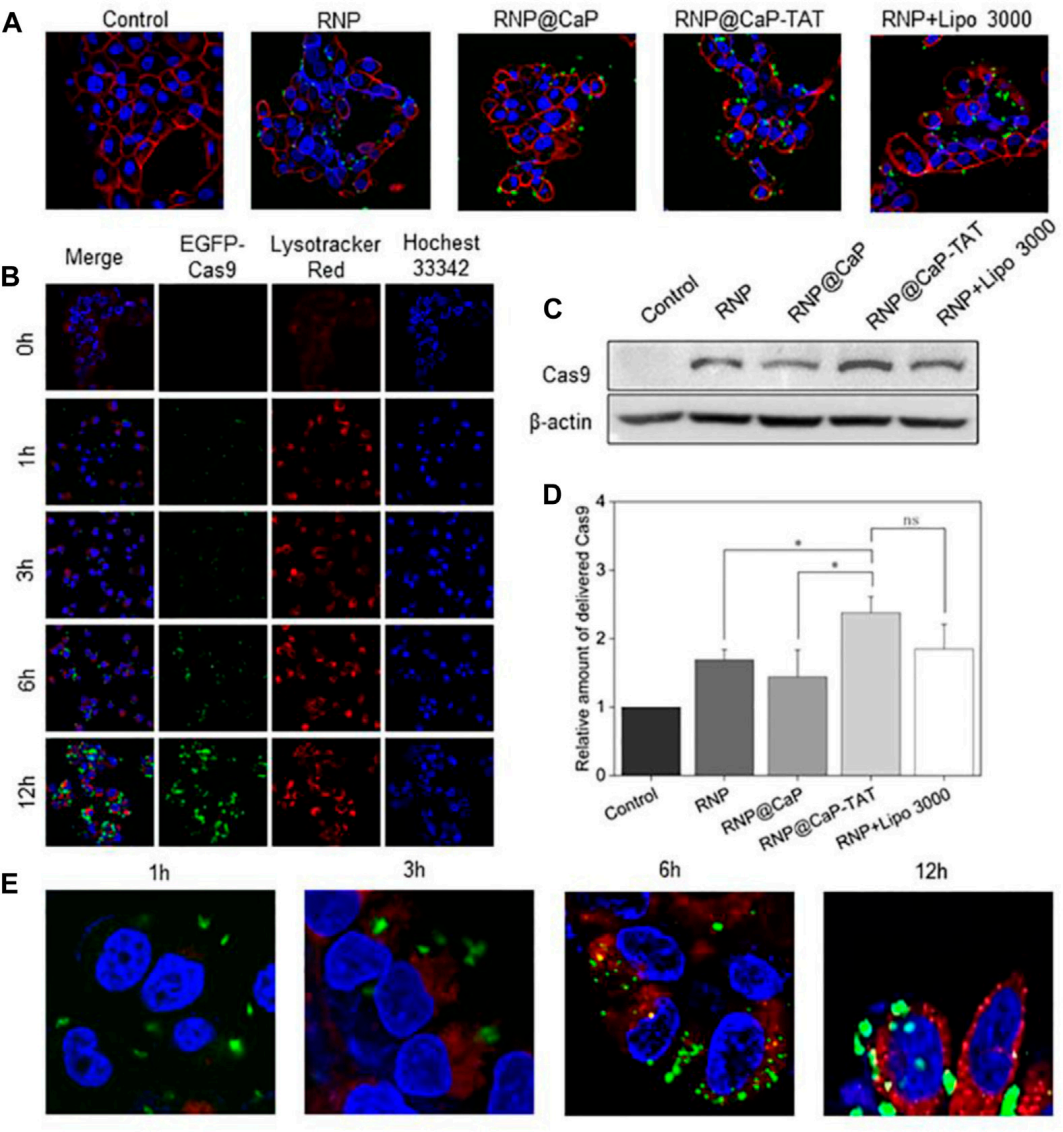
Cellular internalization of RNP formulations monitored by confocal microscopy. **(A)** CLSM images (40×) of PC-3 cells after incubation with RNP, RNP@CaP, RNP@CaP-TAT and RNP + Lipo 3000 (green) for 6 h. WGA was used to stain the cytomembrane (red). **(B)** PC-3 cells were treated with RNP@CaP-TAT for 0, 1, 3, 6 and 12 h, respectively. Blue, nucleus; Green, EGFP-Cas9; Red, acidic organelles (endosomes). **(C)** Western Blot analysis of RNP proteins in PC-3 cells. **(D)** Relative expression level of key proteins in **(C)**; *p < 0.05 compared with the control. **(E)** CLSM images (40×) of PC-3 cells after incubation with RNP@CaP-TAT (green) for 1, 3, 6 and 12 h. Lysotracker Red (red) was used to stain the acidic organelles (endosomes). The merged images (yellow) were used to confirmed that RNP@CaP-TAT was released and delivered into the endosomes within 12 h of uptake.

Since the target sites of Cas9 RNPs are in the nucleus, the endocytosis of RNP@CaP-TAT was further examined *in vitro*. Cas9 with the green fluorescent protein EGFP was used to construct Cas9 RNP, RNP@CaP and RNP@CaP-TAT, and were cocultured with CRPC cell line PC-3 for 6 h. The locations of nanoparticles with EGFP and cell membrane stained with WGA (red fluorescence) were observed using fluorescence microscopy at 0, 1, 3, 6 and 12 h, respectively. As shown in Figure 2A, free RNP could not penetrate through the cell membrane, so there is less significant green fluorescent can be seen in the cells. And RNP@CaP-TAT were significantly superior to the RNP@CaP group, which was as efficient as comparable to the transfection ability of the commercial Lipofectamine 3000 reagent. This result exactly confirmed our previous assumption that the addition of calcium and TAT could improve the ability of Cas9 RNP to enter cells, and the significant effect can be observed after the addition of TAT (Supplementary Figure S7). Western blot analysis was basically consistent with the result of confocal fluorescence microscopy (Figures 2C, D).

Furthermore, we evaluated the transport capacity of RNP@CaP-TAT to deliver Cas9 protein. The nanoparticle demonstrated efficient cytosolic delivery of RNP to PC-3 cells (Figure 2B). After 6 h of incubation, Cas9-EGFP protein was observed to disperse in the nucleus and gradually increased with time.

We also investigated its intracellular delivery by tracing the fluorescence of RNP@CaP-TAT (EGFP, green) in cells. RNP@CaP-TAT (Green) and Lysotracker (red) observed yellow fluorescence co-localization images of cells at 3–6 h. The results of the quantitative analysis indicating that RNP is delivered intracellular through the lysosomal pathway by Image J, but co-localization coefficiency was 0.43, which might be due to the instantaneity of the pH response (Figure 1D). Finally, green fluorescence was observed at 6–12 h in the nucleus (Hocehst 33342, blue) as evidence for the entry of RNP into the nucleus (Figure 2E). In conclusion, the nanoparticle can be effectively taken up by PC-3 cells and deliver RNP to the nucleus for gene editing effect.

### 3.4 The *EphA2* genome-editing effects of RNP@CaP-TAT

To further validate RNP@CaP-TAT genome-editing efficiency, we investigated the intracellular delivery of RNP@CaP-TAT targeting *EphA2* in PC-3 cells. Based on the evidence that Cas9 RNP can enter the nucleus, we assessed the extent of RNP-targeted DNA cleavage and repair drives INDELs *via* the endogenous NHEJ pathway.

**FIGURE 3:**
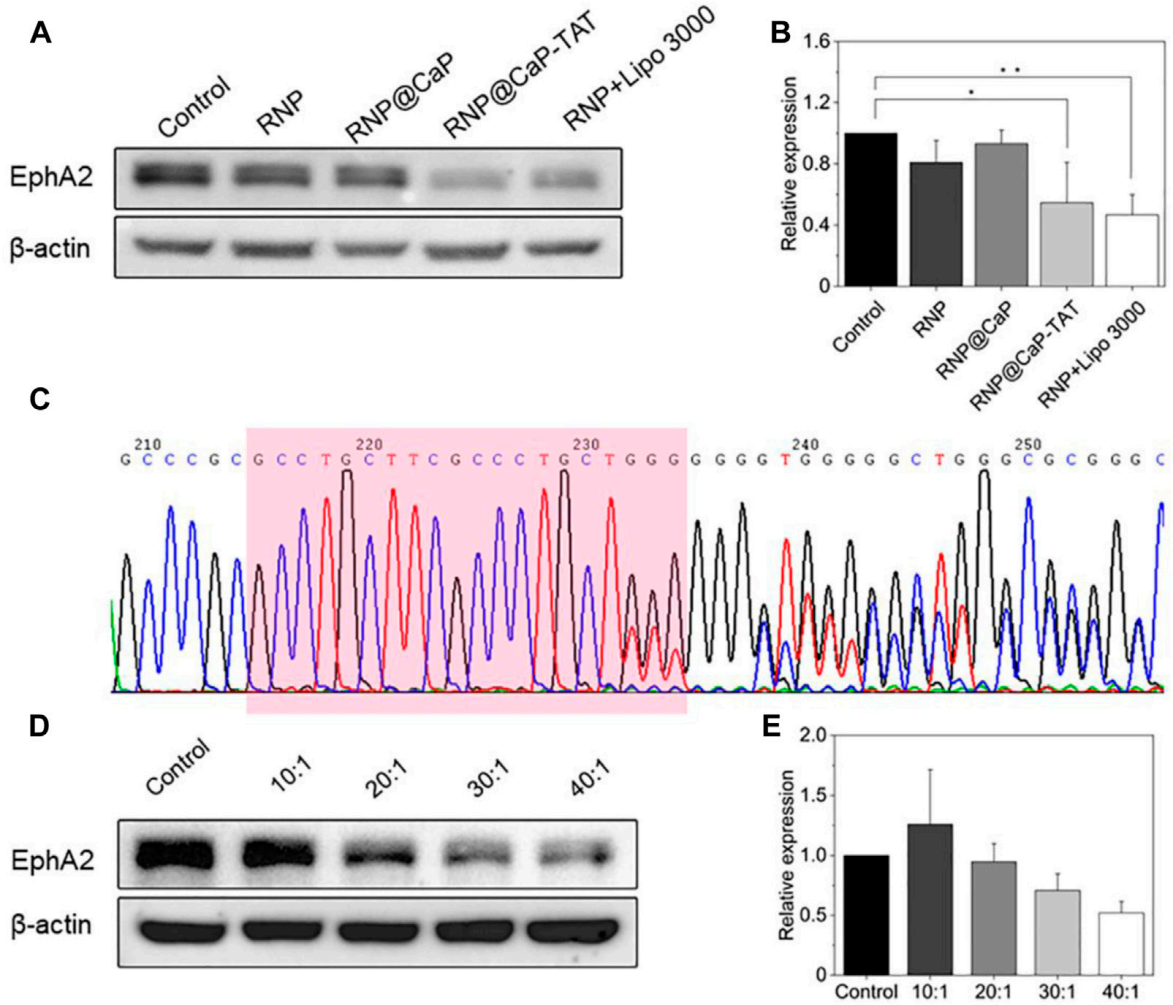
**(A)** Western blot assay for analyzing the EphA2 expression in PC-3 cells treated by RNP, RNP@CaP, RNP@CaP-TAT and RNP + Lipo 3000. **(B)** Relative expression level of EphA2 proteins in **(A)**; *p < 0.05, **p < 0.01 compared with the control. **(C)** DNA Sanger sequencing map after cell transfection. Downstream of the target sequence (marked in pink box), it shows small miscellaneous peaks with irregularity besides a single peak, indicating that on-target site cleavage by CRISPR system were induced. **(D,E)** Western blot assay with different Ca^2+^/RNP quantity ratios.

In response to the differences in the entry of different components into the cytoplasm, we transfected PC-3 cells with RNP@CaP-TAT and determine whether Cas9 RNP inhibit the expression of *EphA2* protein through Western blot. Compared with other groups, the expression of *EphA2* protein induced by RNP@CaP-TAT was significantly down-regulated by about 48% (Figures 3A, B), which was consistent with the results of cell endocytosis experiments. To further determine the rationality of nanoparticle selection, we transfected PC-3 cells with different proportion groups in the synthesis process and evaluated their biological effects by Western blotting. The genomic knockout efficiency is still consistent with the stability of DLS particle size (Figures 3D, E), which proves again that RNP@CaP-TAT is the optimal structure for delivering RNP.

To further confirm the knockout efficiency, the genomic DNA of the cells transfected with RNP@CaP-TAT was extracted, and the linear DNA fragment containing the target sequence was amplified by PCR. DNA sequencing was performed by Sanger method, the sequencing results were shown in Figure 3C. After PC-3 cells transfected with RNP@CaP-TAT, significant overlapping peaks were shown in the target and the downstream sequence of the target due to the frame shift mutation, which caused by the deletion or insertion of bases in the gene repair of the target region. Therefore, Sanger sequencing revealed that RNP@CaP-TAT could effectively knockout the *EphA2* gene in PC-3 cells.

### 3.5 Biological effects of genome editing of RNP@CaP-TAT

**FIGURE 4:**
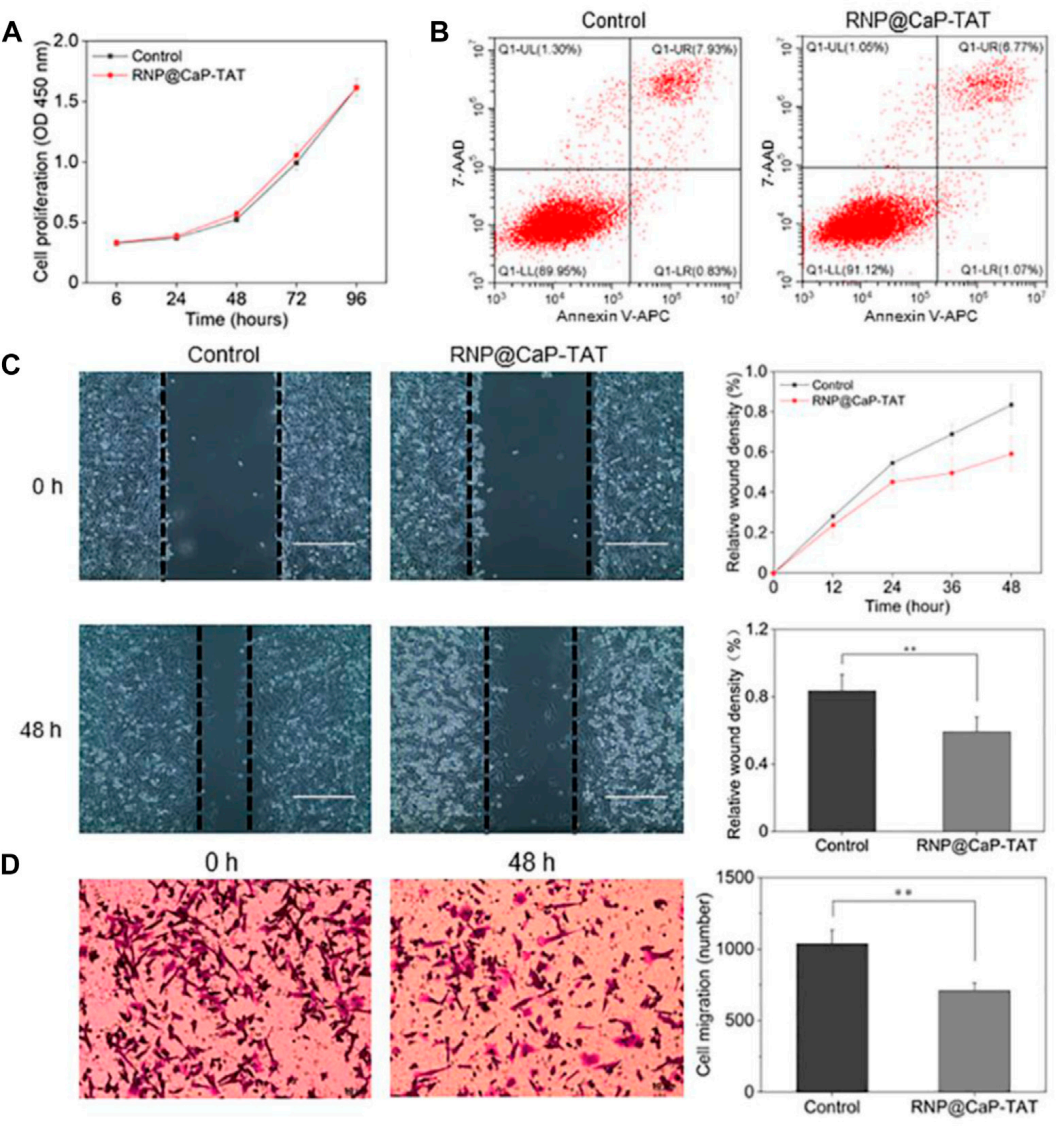
**(A)** Tumor cell proliferation analysis using the CCK-8 assay. **(B)** Analysis of cellular apoptosis using flow cytometry. PC-3 cells were stained using Annexin V-APC and 7-AAD. **(C)** Typical images and migration area of the wound-healing assay. **(D)** Typical images of the cell invasion and the number of invaded cells with the transwell assay. All data are represented as the means ± SD from three independent experiments: **p < 0.01.

Biological effects of PC-3 cells were examined after knockout of *EphA2*. The CCK-8 assay demonstrated that there was no significant difference between the cells in the control group and in the treatment group (treated with RNP@CaP-TAT) (Figure 4A). In addition, cellular apoptosis was analyzed by flow cytometry as presented in Figure 4B. The proportion of early apoptosis (region Q1-LR) in the RNP@CaP-TAT-treated cells slightly increased compared with that in the RNP@CaP-TAT-control cells (0.83% vs. 1.07%). However, there was no statistically significance between the two groups (*p* > 0.05).

Next, the wound-healing assay (Figure 4C) and the transwell invasion assay (Figure 4D) were used to assess tumor cell migration and invasion in each treatment group *in vitro*. Compared with untreated cells, the co-incubation of RNP@CaP-TAT with PC-3 cells caused a significant inhibition of cell migration, as shown in Supplementary Figure S8, and the invasive ability of PC-3 cells transfected with RNP@CaP-TAT was also significantly inhibited. These results show that the Cas9 RNP delivery system can effectively inhibit cell migration and invasion, not cell proliferation and apoptosis *in vitro*.

## 4 Discussion

Non-viral vectors have been widely studied to the delivery of CRISPR/Cas9 gene editing system, compared with the viral vectors, based on their satisfied biocompatibility and high loading rate, which can achieve considerable knockout efficiency. However, the cytotoxicity, metal residues and the complex synthesis process remain controversial. Therefore, how to deliver CRISPR/Cas9 in a simple, and safe effective way is essential. In addition, castration-resistant prostate cancer (CRPC) is a progressive stage of prostate cancer with no effective treatment at present. As a complicated disease, the initiation and progress of cancer involves multiple genetic mutations, so gene therapy undoubtedly provides new ideas for prostate cancer treatment. And prostate cancer is highly applicable to gene therapy, due to its physiological location and pathological basis. However, few studies on non-viral vector delivery of CRISPR/Cas9 to treat CRPC were reported.

Our synthesis strategy focus on the simplicity of the preparation process and high biocompatibility, which meet the practical needs of non-viral delivery of gene editing systems. Calcium phosphate core and TAT peptide are thought as potential, useful vectors components for gene therapy because of their low cytotoxicity. CPP has been used to deliver various anticancer drugs to cancer cells *in vivo* and has been observed to effectively inhibit tumor growth in preclinical mouse models. Therefore, our study examined the safety and transfection efficiency of RNP@CaP-TAT complexes *in vitro* and investigated whether gene delivery could produce effective biological effects *in vitro*. In summary, we developed a genome-editing nano-assembly targeting *EphA2* through delivery of Cas9 RNP, which may break through the limitation of the current clinical treatments in patients with castration-resistant prostate cancer (CRPC). The electrostatic interaction greatly simplifies the preparation process of this new vector, and its high loading rate can maximize the use of Cas9 RNP for gene editing with good biocompatibility and the *EphA2* gene knockout efficiency up to 48%, which can effectively inhibit the migration of PC-3 cells.

However, we still have some limitations. Less image positioning was captured in the process of lysosome co-localization, and the reason may be the instantaneity of lysosome escape. This result is consistent with the *in vitro* simulation of pH responsiveness (hydrated particle size results). And another reason may be that calcium phosphate nanoparticles tend to accumulate in the cytoplasm affecting the endocytosis efficiency of nanocarriers. Based on the above, the stability of calcium phosphate nanoparticles needs to be further optimized, as it is directly related to CRISPR/Cas9 gene editing efficiency. In addition, further studies are needed to verify the biological effects *in vivo* to deepen the research significance of this preliminary exploration. Overall, this study demonstrates that targeting *EphA2* by genome-editing nano-assemblies can become a potential therapeutic strategy for the treatment of CRPC, and our approach will offer new insights to design CRISPR/Cas9-based nanomedicine for gene therapy, especially in the application of other tumors with high *EphA2* expression.

## Data Availability

The original contributions presented in the study are included in the article/Supplementary Materials, further inquiries can be directed to the corresponding authors.
