# Supplementary material for: Calcium phosphate-based nanomedicine mediated CRISPR/Cas9 delivery for prostate cancer therapy
Source: Front Bioeng Biotechnol. 2022 Dec 14;10:1078342. doi: 10.3389/fbioe.2022.1078342 (PMC9794984; doi:10.3389/fbioe.2022.1078342)
Supplement: Supplementary file 1 [file DataSheet1.docx]

Supplementary Material

# Supplementary Figures

**
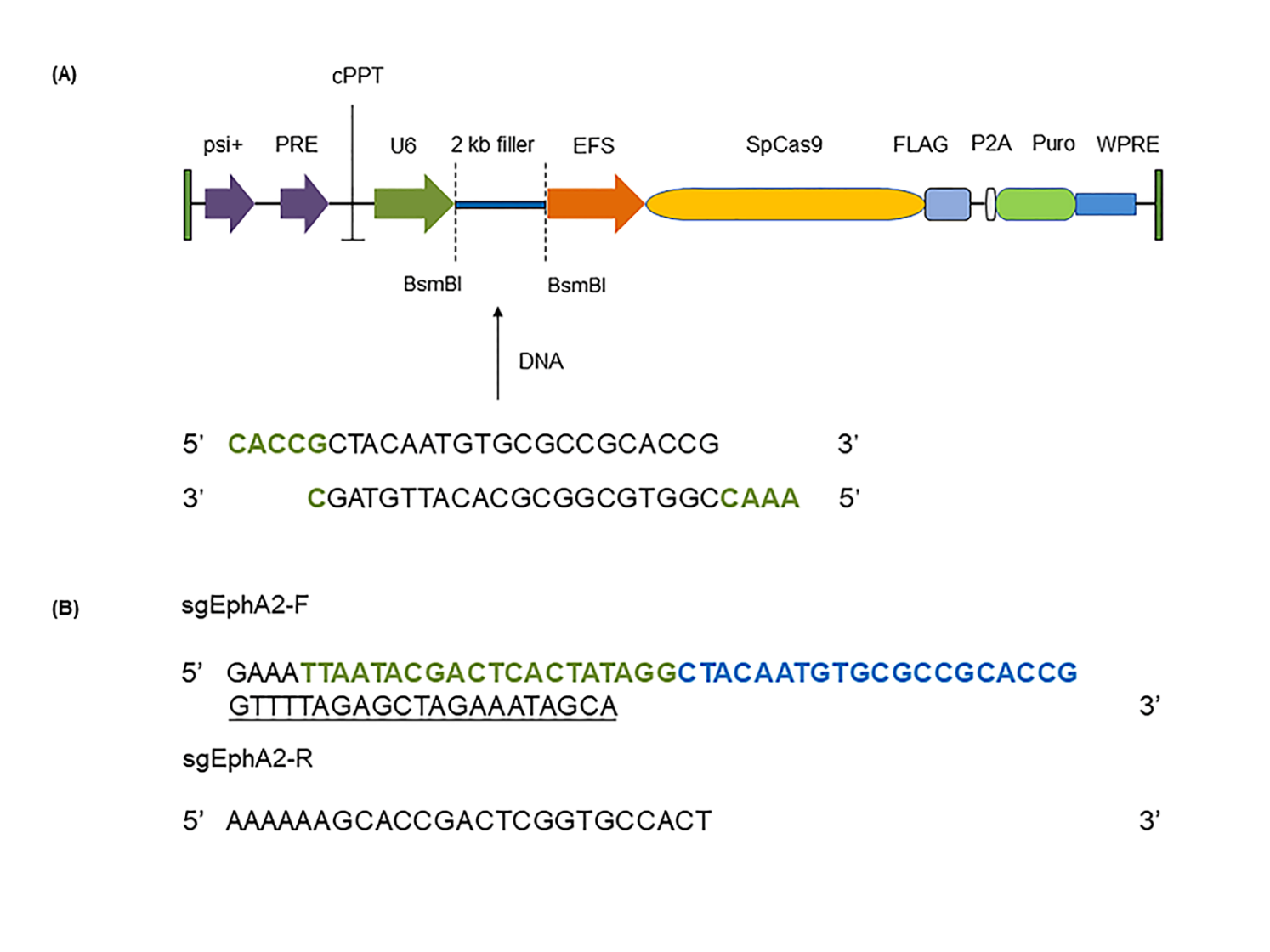
**

**Figure S1**. (A) Schematic map of lentiCRISPRv2. Green: BsmBI restriction endonuclease cutting site. (B) Sequence of the PCR primers to linearize the sgRNA transcription template. Green: T7 transcriptase recognition site (T7 promoter). Blue: gRNA sequence targeting EphA2.Underline: crRNA

**
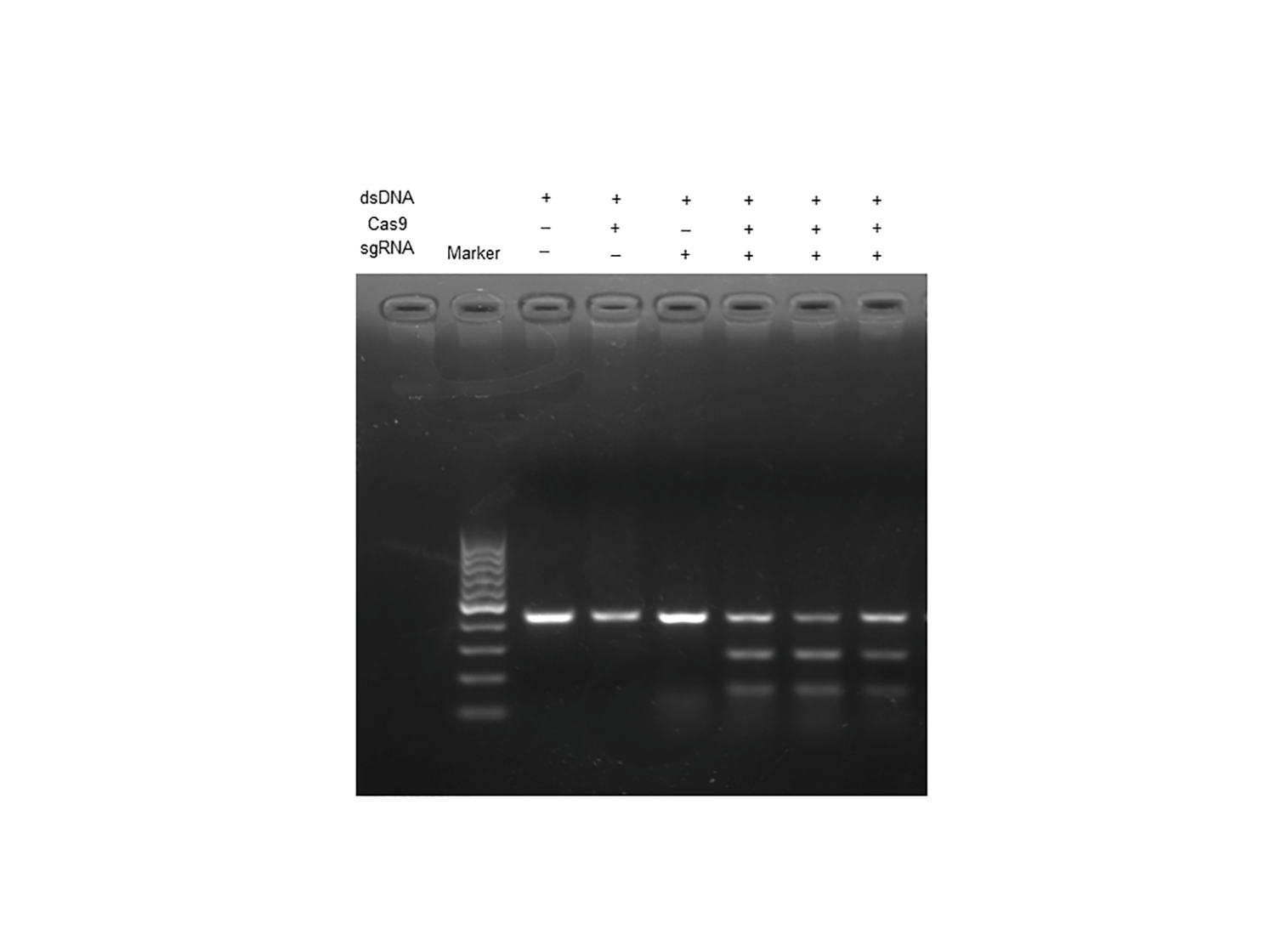
**

**Figure S2.** Gel electrophoresis analysis of the RNP formulations.

**
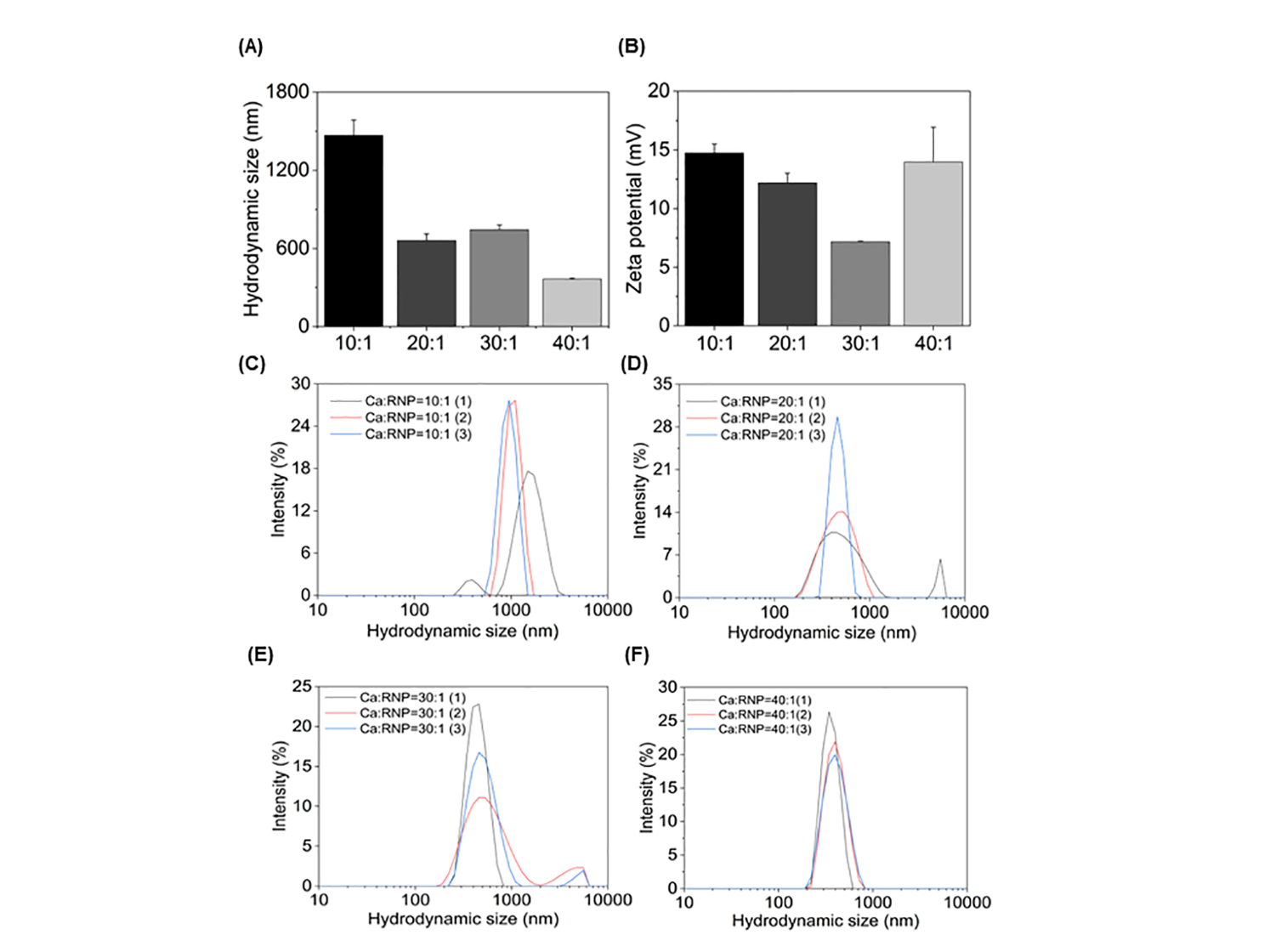
**

**Figure S3.** (A, C-F) Hydrodynamic particle size distribution of different Ca^2+^/ RNP quality ratios. (C) 10:1 (D) 20:1 (E) 30:1 (F) 40:1. All the quantitative data represent mean ± SD (n = 3)

**
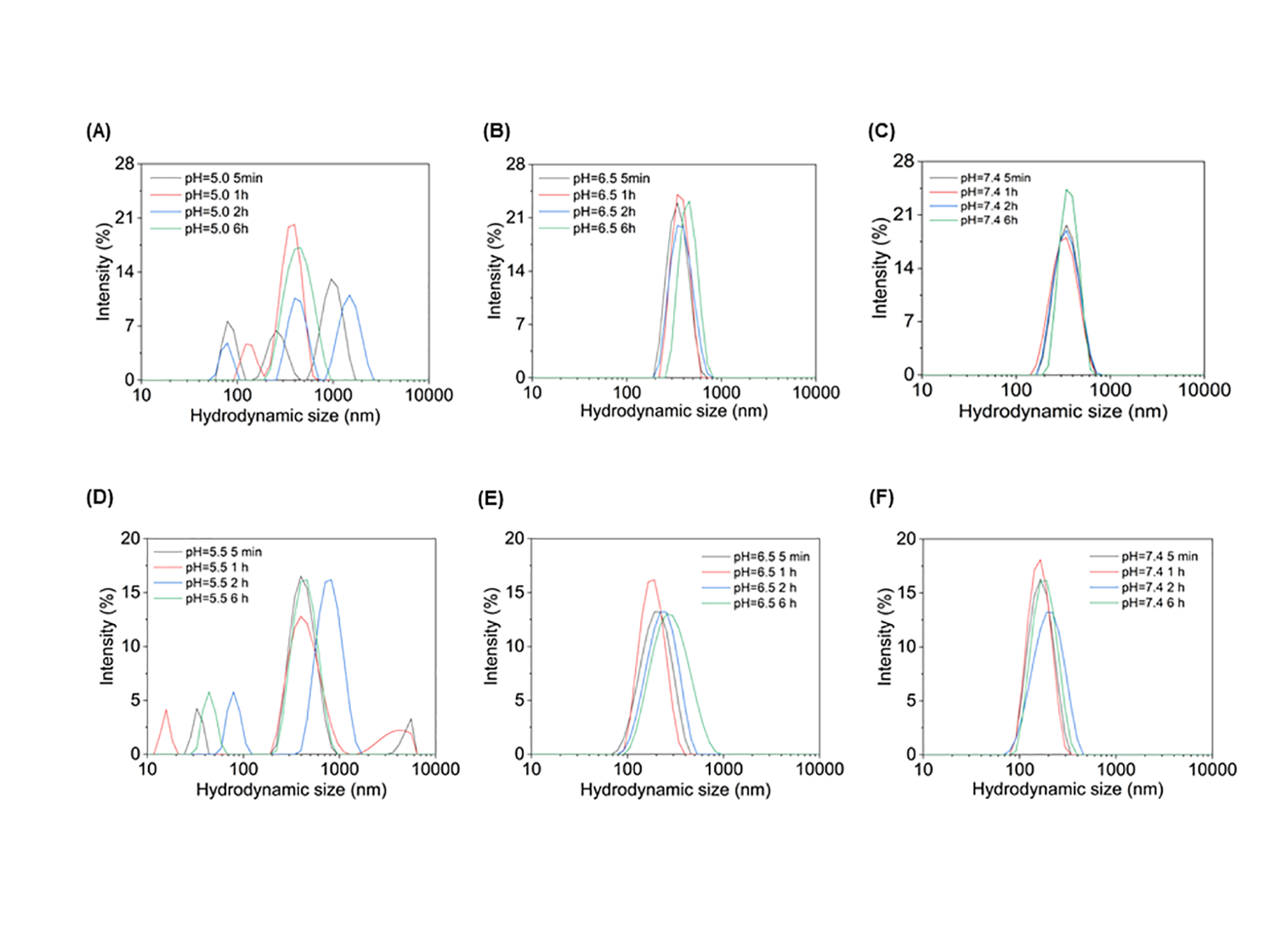
**

**Figure S4.** Hydrodynamic particle size distribution of the different pH in acetate buffer solutions (A-C) and cell culture medium (D-E).


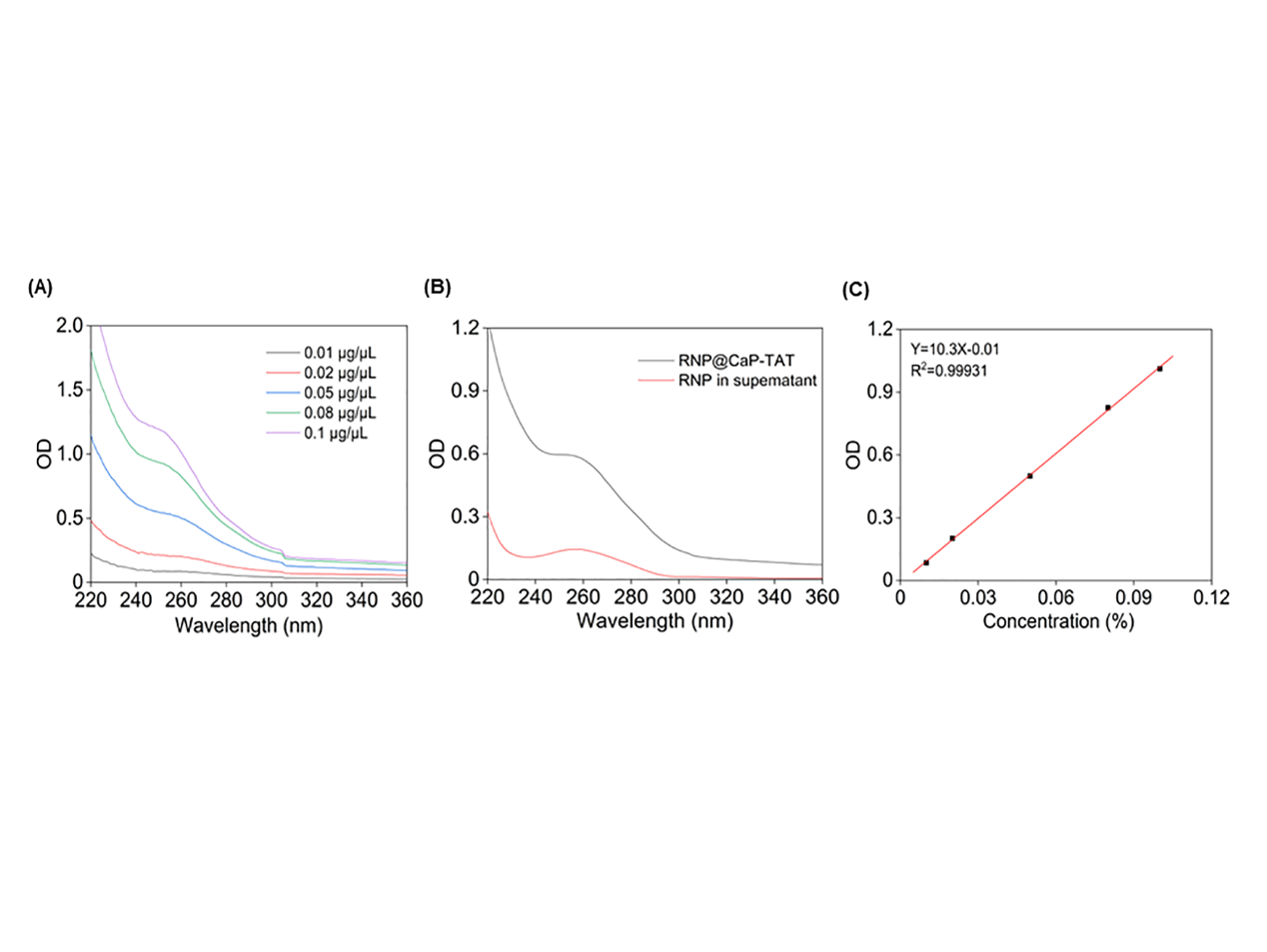


**Figure S5.** The Cas9 RNP loading rate of RNP@CaP-TAT

**
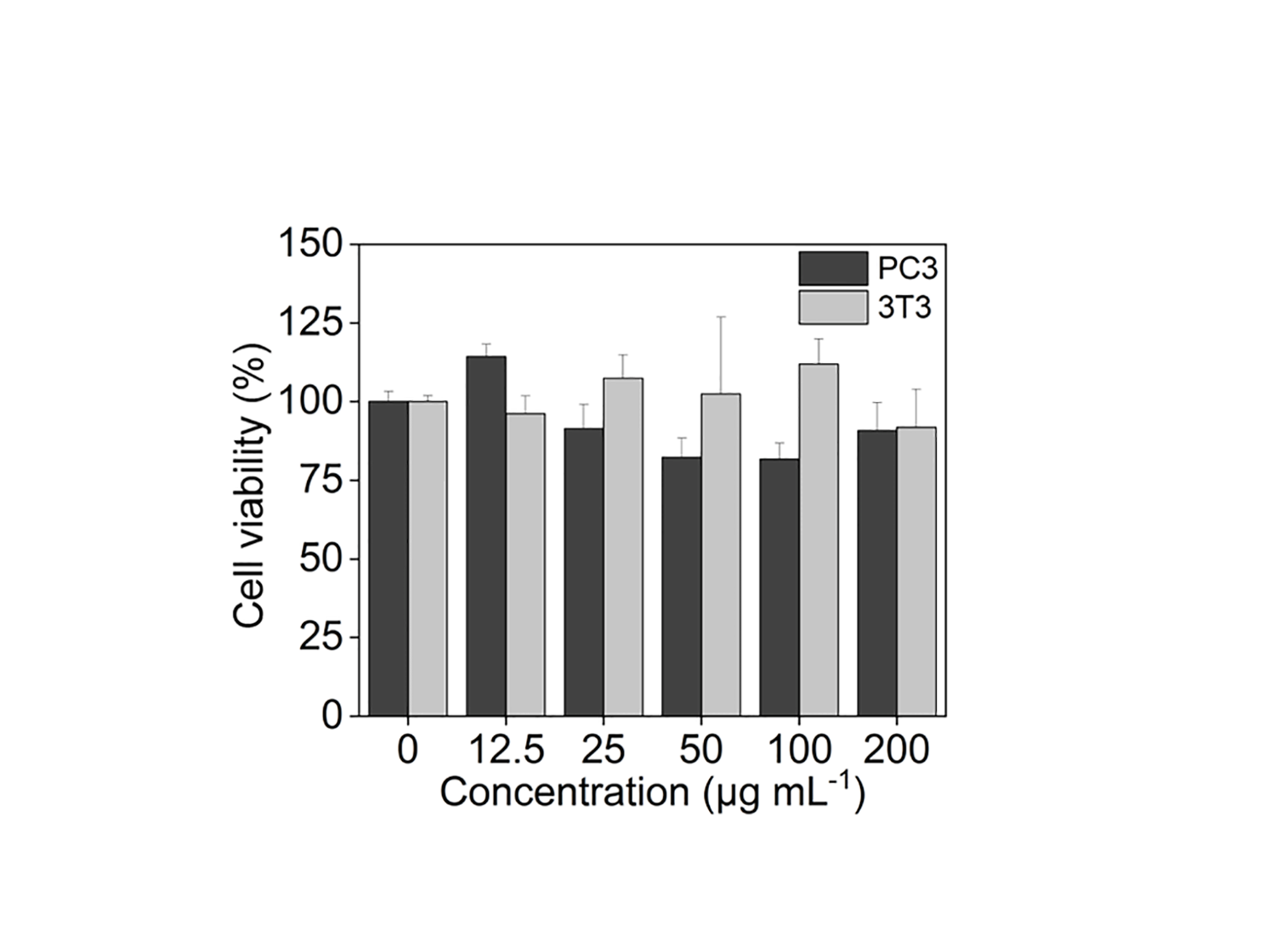
**

**Figure S6.** Cell viability of PC-3 and 3T3 cells after the treatment with different concentrations of RNP@CaP-TAT nanoparticles for 48 h, respectively. All the quantitative data represent mean ± SD (n = 3)


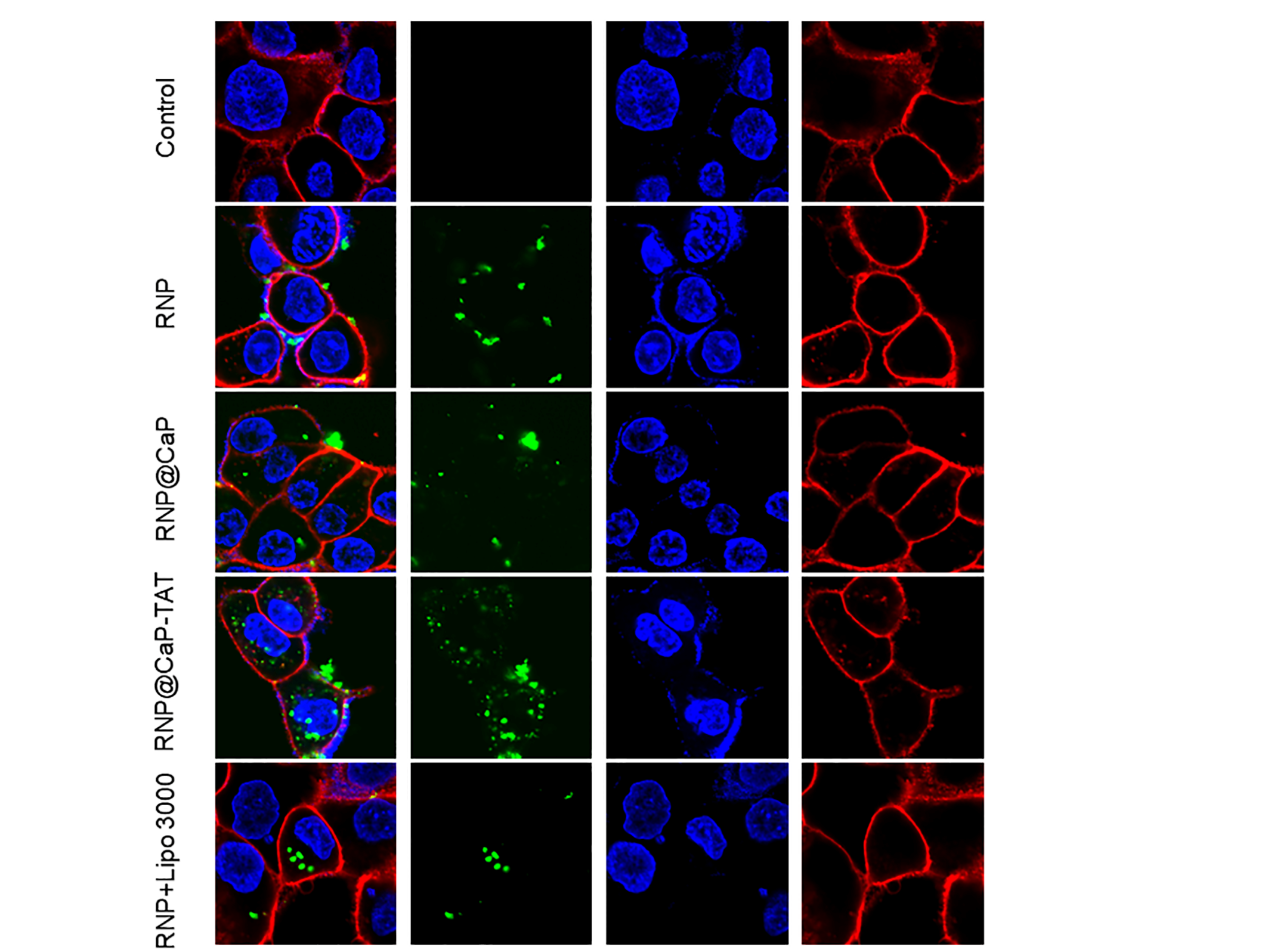


**Figure S7.** Cellular internalization of RNP formulations monitored by confocal microscopy. The cytomembrane were stained with WGA, and the cell nuclei were stained by Hoechst 33342. The cells were incubated with the nanoparticles for 6 h.

**
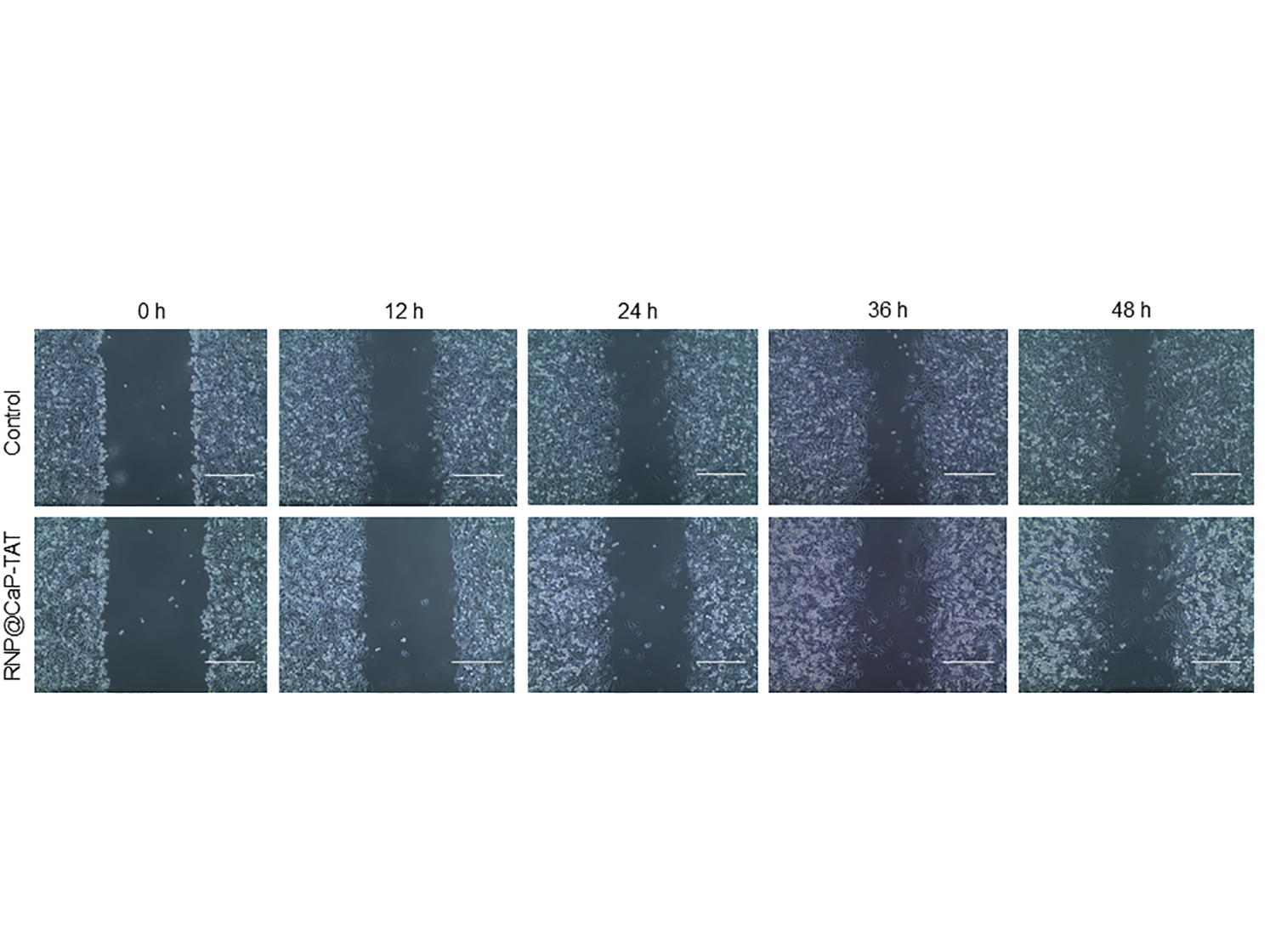
**

**Figure S8.** Wound healing assay.
